# Digital Technology to Deliver a Lifestyle-Integrated Exercise Intervention in Young Seniors—The PreventIT Feasibility Randomized Controlled Trial

**DOI:** 10.3389/fdgth.2020.00010

**Published:** 2020-07-31

**Authors:** Kristin Taraldsen, A. Stefanie Mikolaizak, Andrea B. Maier, Sabato Mellone, Elisabeth Boulton, Kamiar Aminian, Clemens Becker, Lorenzo Chiari, Turid Follestad, Brenda Gannon, Aniosora Paraschiv-Ionescu, Mirjam Pijnappels, Ingvild Saltvedt, Michael Schwenk, Chris Todd, Fan B. Yang, Anna Zacchi, Jeanine van Ancum, Beatrix Vereijken, Jorunn L. Helbostad

**Affiliations:** ^1^Department of Neuromedicine and Movement Science, Norwegian University of Science and Technology (NTNU), Trondheim, Norway; ^2^Department of Clinical Gerontology, Robert Bosch Krankenhaus, Stuttgart, Germany; ^3^Department of Human Movement Sciences, Vrije Universiteit Amsterdam, Amsterdam, Netherlands; ^4^Department of Medicine and Aged Care, @AgeMelbourne, The University of Melbourne, The Royal Melbourne Hospital, Melbourne, VIC, Australia; ^5^Department of Electrical, Electronic and Information Engineering ≪Guglielmo Marconi≫, University of Bologna, Bologna, Italy; ^6^School of Health Sciences, Manchester Academic Health Science Centre, The University of Manchester, Manchester, United Kingdom; ^7^Laboratory of Movement Analysis and Measurement, Ecole Polytechnique Federale de Lausanne, Lausanne, Switzerland; ^8^Centre for Business and Economics of Health, The University of Queensland, Brisbane, QLD, Australia; ^9^Department of Geriatrics, Clinic of Medicine, St Olavs hospital, University Hospital of Trondheim, Trondheim, Norway; ^10^Manchester University NHS Foundation Trust, Manchester, United Kingdom; ^11^Centre for Health Economics, University of York, York, United Kingdom; ^12^Doxee s.p.a., Modena, Italy; ^13^CINECA, Bologna, Italy

**Keywords:** physical activity, muscle strength, balance, behavioral change, mHealth

## Abstract

**Background:** Behavioral change is the key to alter individuals' lifestyle from sedentary to active. The aim was to assess the feasibility of delivering a Lifestyle-integrated Functional Exercise programme and evaluate the delivery of the intervention by use of digital technology (eLiFE) to prevent functional decline in 61–70 year-old adults.

**Methods:** This multicentre, feasibility randomized controlled trial was run in three countries (Norway, Germany, and the Netherlands). Out of 7,500 potential participants, 926 seniors (12%) were screened and 180 participants randomized to eLiFE (*n* = 61), aLiFE (*n* = 59), and control group (*n* = 60). eLiFE participants used an application on smartphones and smartwatches while aLiFE participants used traditional paper-based versions of the same lifestyle-integrated exercise intervention. Participants were followed for 12 months, with assessments at baseline, after a 6 month active trainer-supported intervention, and after a further 6 months of unsupervised continuation of the programme.

**Results:** At 6 months, 87% of participants completed *post-test*, and 77% completed the final assessment at 12 months. Participants were willing to be part of the programme, with compliance and reported adherence relatively high. Despite small errors during start-up in the technological component, intervention delivery by use of technology appeared acceptable. No serious adverse events were related to the interventions. All groups improved regarding clinical outcomes over time, and complexity metrics show potential as outcome measure in young seniors.

**Conclusion:** This feasibility RCT provides evidence that an ICT-based lifestyle-integrated exercise intervention, focusing on behavioral change, is feasible and safe for young seniors.

**Clinical Trial Registration:** ClinicalTrials.gov, identifier: NCT03065088. Registered on 14 February 2017.

## Introduction

Western societies mostly enjoy a steadily increasing life expectancy (1), but the majority of older citizens spend their life with morbidities and disabilities that ultimately require care (2). The pace of this change and its social, economic and health-care consequences have not been adequately addressed (3). A shift in focus from treatment toward prevention of age-related diseases and disability, by promoting active and healthy aging, is highly warranted (4), but effective strategies are yet to be established.

Behavioral change is the key to alter individuals' lifestyle from sedentary to active. However, long-term adherence to physical activity interventions is challenging, with activity levels typically reverting back to previous low levels after an intervention (5, 6). There is an imperative to develop effective strategies promoting physical activity, which are acceptable and sustainable over the long term. Activity programmes that are multi-dimensional and integrated into daily life have shown to positively influence habit formation, improve function and decrease disability when compared to “traditional” exercise that are segregated from daily routines (7). Integrating tailored activities into daily life has been shown to be effective in adults aged over 70 years, however this approach has not yet been adopted to prevent accelerated functional decline in young seniors aged between 61 and 70 years.

Information and communication technology (ICT) is increasingly used to deliver healthcare and behavioral change interventions (8–10). These electronic health interventions (eHealth) make use of electronic devices such as computers, smartphones or smartwatches to deliver or assist interventions. The digital environment enables feedback and tailored interventions at an individual level (9, 11, 12). eHealth interventions for promoting an active lifestyle, including an increase in physical activity, are promising and show a positive short-term effect on physical activity, but long-term effects on sustained behavioral change have yet to be established (9).

The PreventIT project is a European Horizon 2020 Personal Health and Care project, aimed to develop and test a personalized behavior change intervention on physical activity for young seniors at risk for accelerated functional decline (13). The original Lifestyle-integrated Exercise (LiFE) programme developed by Clemson et al. (7) was adapted to the needs of young seniors, tailoring exercise at an individual level and integrating it into daily life, delivered by either a traditional paper-based *adapted* manual (aLiFE) or *enhanced* by an eHealth smartphone and smartwatch-based system (eLiFE).

The primary aim was to test the feasibility of integrating physical activities into daily life of young seniors aged 61–70 years by the aLiFE or eLiFE programme, compared to a control group who received general written physical activity recommendations only. To evaluate and further improve the intervention for a future phase III clinical trial, the evaluation addressed participation and adherence; feasibility and usability of the programmes; acceptability of eLiFE delivered using smartphone and smartwatch technology; estimates of change in function and physical activity; and feasibility of health economics evaluation.

## Methods

The PreventIT feasibility randomized controlled trial has been conducted according to the detailed description in the published protocol (13).

### Trial Design and Participants

This three-armed, feasibility randomized controlled trial was ethically approved and was run from March 2017 until August 2018 at three clinical sites: Trondheim, Norway (REK midt, 2016/1891), Stuttgart, Germany (registration number 770/2016BO1), and Amsterdam, The Netherlands (registration number 2016.539; Dutch Trial Registry NL59977.029.16). Participants were recruited via invitation letters, which were sent to a random sample of individuals born between 01/01/1947 and 31/12/1956, drawn from the respective local population registries. Invitation letters were sent, and responders were screened for eligibility. All participants provided written informed consent prior to commencing the on-site assessments.

### Screening Procedures

Initial screening consisted of a telephone interview assessing the following eligibility criteria: aged 61–70 years, retired or working part-time, community dwelling (living independently), able to read a newspaper or text on a smartphone, speak Norwegian/German/Dutch, able to walk 500 meters without a walking aid, and available for home visits during the following 6 weeks. Those already participating in an organized exercise class (>1/week), undertaking moderate-intensity physical activity (≥150 min/week in the previous 3 months), or with long-term travel plans (>2 months) within the next 6 months were excluded. A web-based risk screening tool (14) was used to describe participants' risk of long-term accelerated functional decline. Medical screening ensured that exercise was not contraindicated. The final exclusion criteria included cognitive impairment (Montreal Cognitive Assessment (MoCA) ≤ 24 points) (15) or depression (defined as acute depression by a health professional at assessment in Trondheim and Amsterdam, as major depression with CES-D (16) cut-off of >24 points in Stuttgart.

### Interventions

Of the three treatment groups, two received an active intervention (aLiFE or eLiFE) using the activity framework (13) and one acted as a control group. In short, the programme consisted of strategies to (a) improve balance, (b) increase muscle strength, and (c) reduce sedentariness and increase physical activity. The aLiFE programme was adapted to young seniors to be more challenging than the LiFE programme. The initial development and evaluation of aLiFE is published elsewhere (17). In addition, the programme comprised a behavioral change framework, aiming to turn activity intentions into a habit by embedding activities into daily life (18).

The eLiFE intervention was delivered to participants via the PreventIT application on a smartphone and a smartwatch through video clips, pictures, and text/verbal instructions for each activity. The eLiFE participants were provided with both an android phone and a smartwatch they could use during the entire study period. aLiFE participants received a paper-based manual with descriptions and instructions for the same activities.

The intervention was delivered actively for 6 months post-randomization. Participants in aLiFE and eLiFE received six and four home visits from trainers, respectively, plus three phone-calls. After the 6 month active intervention period, participants were encouraged to continue with their personalized activity programme for the next 6 months, without the assistance from trainers (unsupervised follow-up).

The control group participants received one home visit entailing a two-page written summary of the WHO recommendations for physical activity (19).

### Sample Size

No sample size calculation was performed for this study, as it is a feasibility study not designed to conclude on effectiveness.

### Randomization

Participants were randomized following the 7-day activity monitoring at baseline (T1), using a web-based randomization procedure developed, used and run by the Clinical Research Unit Central Norway, at NTNU, Norway. The randomization was stratified to clinical sites and performed by block randomization, where block sizes varied. One non-blinded person at each clinical site performed the web-based randomization. Recruitment continued until 60 participants completed the first home visit per study site.

### Blinding

Pre-intervention measures were assessed by trained research and medical staff, prior to randomization. Post-intervention measures were collected by personnel blinded to group allocation. Due to the nature of the intervention, it was not possible to blind participants or the instructors delivering the intervention. Outcome measures that identified group allocation were collected by non-blinded research staff.

### Assessment and Outcome Measures

Participants were assessed at baseline (T1), and 6 (T2) and 12 months (T3) post-randomization. Participation was evaluated based on adherence to the participant's individual intervention protocol. Information regarding the number of home visits/calls received, and trial completion vs. withdrawn were collected. Uptake and adherence to recommendations were assessed monthly via email or post using a single question (13), where each month of reporting was summarized as full adherence (responded positively, and “all or more than planned”), partial adherence (responded positively, and “but not as much as intended”), or non-adherence (responded negatively). Furthermore, the Exercise Adherence Reporting Scale (EARS) (20) was completed by participants during T2 and T3 assessment.

For the feasibility and usability, adverse events and participant progression throughout the trial were documented. Detailed information was collected via questionnaires at T2 and T3 regarding the acceptability of the aLiFE and eLiFE interventions. Participants were asked to report the type and difficulty level of the activities they had integrated into their daily lives. Intervention-specific questions were asked, to ascertain the usefulness of the intervention components (training manuals, home visits, personalisation, etc.) and whether activities had become habitual (Self-Report Behavioral Automaticity Index, SRBAI) (21). Focus group interviews were held after T1 with a random sample of totalling 62 participants (24 aLiFE, 21 eLiFE, and 17 Control).

To assess the technology (PreventIT application), the System Usability Scale (SUS) (22) was used; a higher SUS score indicated better product usability, with scores above 68 implying average product usability (23). The Tele-healthcare Satisfaction Questionnaire (TSQ-WT) (24) was used to evaluate benefit, usability, self-concept, privacy and loss of control, quality of life and wearing comfort. A total score was calculated ranging from 0 (no satisfaction) to 120 (extreme satisfaction). To quantify the programme-specific usability, the application usage and the activities selected within the application are reported.

Estimates of change were calculated for the main outcome measures Late-Life Function and Disability Index (LLFDI) (25, 26) and the physical behavior complexity metric (27, 28). LLFDI assesses function (ability to perform specific activities of daily living) and disability (inability to take part in major life tasks and social roles) in community-dwelling seniors. Behavioral complexity was assessed in the domains of physical activity, sleep and social participation. These data were collected using AX3 sensors from Axivity (+/– 2g, 100 Hz) attached to the participant's lower back and wrist which monitored the physical activity over seven consecutive days.

The estimate of change was also calculated for secondary outcome measures. The measures included data regarding general health and function, medication use, neuropsychological measures and physical function (13).

The health economics were based on healthcare resource use and health-related quality of life (HRQoL). Healthcare resource used by participants was collected at T1, T2, and T3 through a questionnaire developed for this study. HRQoL was assessed by the 5-level EuroQol 5-dimension (EQ-5D-5L) instrument, with higher scores indicating better quality of life (29), and by the Short Form-12 (SF-12) survey (23).

### Data Analysis

A complete data analysis plan was finalized before the T2 assessments started. We developed and ran all analyses blinded to group allocation, before we added the randomization variable. Descriptive statistics and *t*-tests were used to present the data for participation, feasibility and usability, and technology. Continuous data are presented as mean (SD) or median (25- and 75-percentils), and categorical data as counts and percentages. The focus groups were audio recorded, transcribed verbatim and translated into English for thematic analysis.

For the estimates of change, main (LLFDI and physical behavioral complexity metrics) and secondary outcome measures were used to evaluate changes in function from T1 to T3 for eLiFE and aLiFE vs. the control group. Linear mixed-models (LMMs) were used, including factors for time point and study allocation, as well as their interaction, and age and sex as independent variables. Within-participant correlations were accounted for by a participant-specific random intercept. Clinical sites were treated as a fixed rather than random effect due to its low number and were included as an independent variable. Estimates of effect sizes for the differences between the three groups, and for changes within the eLiFE and aLiFE groups, are provided as mean differences for the outcome variables. In case of non-normality, appropriate methods were used. The significance level was set to 0.01 to give some protection against false positive findings due to multiple testing. A priori subgroup analyses were performed for high and low risk of functional decline, and for full, partial and non-adherence.

For economic evaluation, costs are expressed in Euro (€) 2017–18 prices. Health outcomes were measured in quality-adjusted life years (QALYs), estimated using the HRQoL scores generated from responses to the EQ-5D-5L, using the UK (30), German (31), and the Dutch value set (32), with the area under the curve approach and linear interpolation (33). Mean difference in costs and QALYs between groups over the 12-month period were estimated using a seemingly unrelated regression model (34), adjusted for baseline costs and baseline EQ-5D scores. The incremental cost-effectiveness ratio (ICER) and the incremental net health benefit (NHB) were calculated, assuming a cost-effectiveness threshold of €30,000 to €40,000 per QALY. Uncertainty about the intervention being cost-effective was evaluated using probabilistic sensitivity analysis (PSA) and presented using cost-effectiveness acceptability curves (CEACs) (35, 36).

## Results

**Figure 1:**
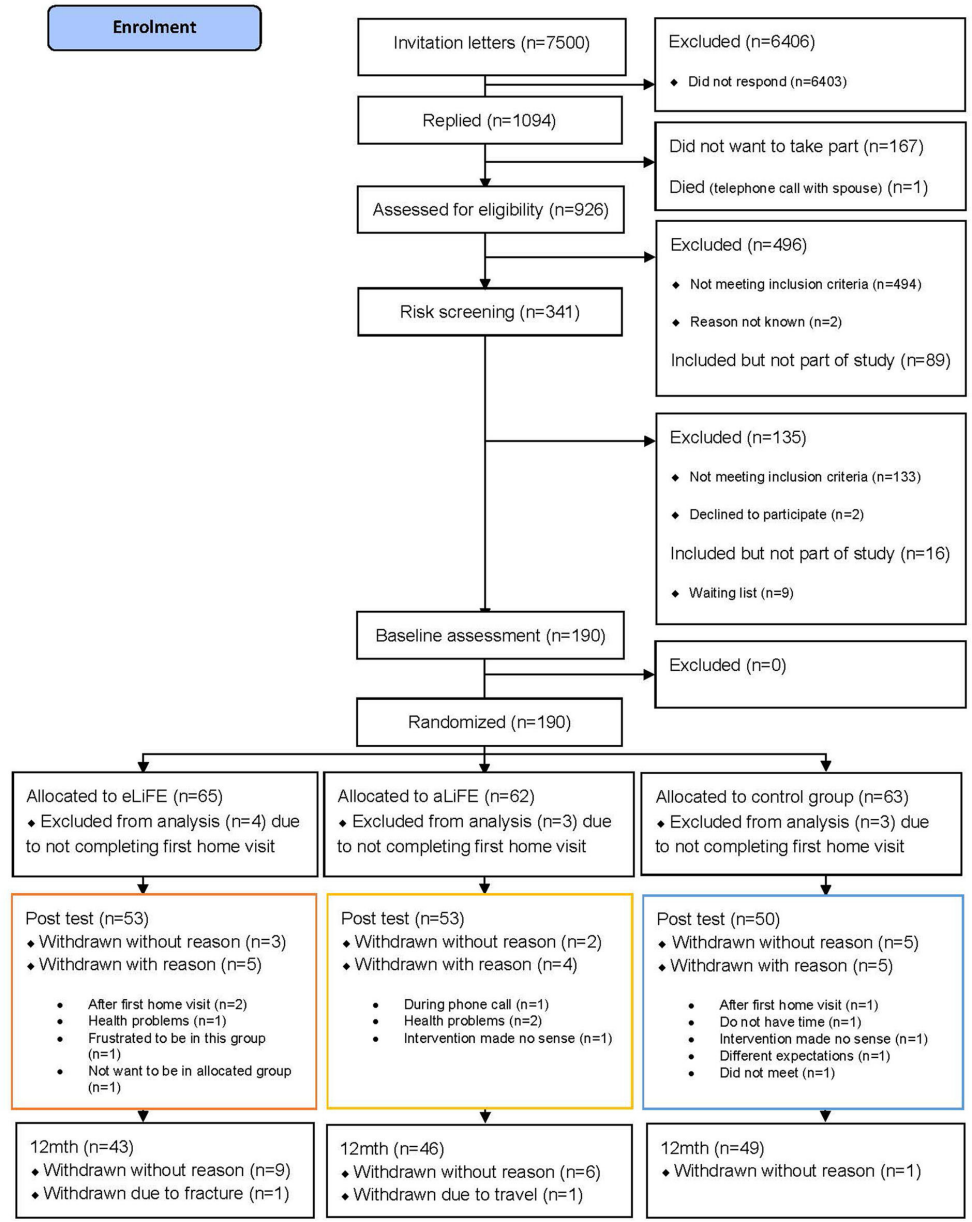
Flow chart.

In total 7,500 invitation letters were sent by mail to seniors between 61 and 70 years of age (2000 in Trondheim, 1500 in Stuttgart, and 4000 in Amsterdam). Following the three-step screening process, 180 participants were successfully enrolled into the study, accepted randomization and completed their first home visit (*n* = 61 eLiFE, *n* = 59 aLiFE, *n* = 60 controls). Baseline characteristics of participants completing the trial (*n* = 138) vs. non-completers (*n* = 47) were not statistically significantly different. The flow of participants from recruitment to trial ending is shown in Figure 1.

### Participant Characteristics

Table 1 presents the participants characteristics at baseline (T1) for the three groups. Mean age for eLiFE (54.1% women), aLiFE (50.8% women), and controls (51.7% women), were 66.4 (SD 2.3), 66.2 (SD 2.3), and 66.4 (SD 2.7) years, respectively. In total, 152 (84.4%) participants reported no falls in the past 6 months. The medication usage and reported diseases were 2 (median numbers), whereas 29.4% reported no medication use. The majority of participants (*n* = 139, 77.2%) had a low risk of functional decline based on the risk screening tool. Preferred gait speed was 1.4 m/sec.

**Table 1:** Participant characteristics.

	**aLiFE**	**eLiFE**	**Control**
	***n* = 59**	***n* = 61**	***n* = 60**
Age, years, mean (SD)	66.19 (2.32)	66.43 (2.33)	66.4 (2.71)
Gender, female, *n* (%)	30 (50.8%)	33 (54.1%)	31 (51.7%)
Height, cm, mean (SD)	171.95 (9.04)	169.77 (9.43)	170.95 (9.17)
Weight, kg, mean (SD)	81.29 (17.24)	80.10 (16.24)	78.42 (16.05)
Living alone, *n* (%)	21 (63.8%)	18 (70.5%)	22 (63.3%)
Pain during rest, 0–10, median (25–75-perc.)	1.0 (1.0–3.0)	1.0 (1.0–3.0)	2.0 (1.0–4.0)
Pain during walking, 0–10, median (25–75-perc.)	2.5 (1.0–4.0)	2.0 (1.0–4.5)	2.0 (1.0–4.0)
**Falls in past year** ***n*** **(%)**			
0	53 (91.4%)	51 (83.6%)	47 (78.3%)
1	4 (6.9%)	10 (16.4%)	6 (10.0%)
2+	1 (1.7%)	0 (0)	7 (11.7%)
**Economic satisfaction**, ***n*** **(%)**			
Good	23 (39.7%)	23 (37.7%)	27 (45.0%)
Sufficient	22 (37.9%)	31 (50.8%)	23 (38.3%)
Poor/bad	13 (22.4%)	7 (11.5%)	10 (16.7%)
Total number comorbidities, median (25–75-perc.)	2.0 (1.0–4.0)	2.0 (1.0–3.0)	2.0 (1.0–3.0)
Total number medication, median (25–75-perc.)	2.0 (1.0–4.0)	2.0 (1.0–3.0)	2.0 (1.0–4.0)
Diagnosis of Arthritis*n (%)	19 (32.8%)	18 (29.5%)	20 (33.3%)
Diagnosis of Cardio-vascular disease*n (%)	9 (15.5%)	14 (23.0%)	(30.0%)

### Feasibility

#### Home Visits/Calls

All participants in the control group (*n* = 60) completed their home visit and 93 of the 120 intervention participants (77.5%) completed all scheduled home visits and phone calls during the 6 months intervention period (79% eLiFE/76% aLiFE). Two out of 27 participants who did not complete all scheduled visits/calls completed the first home visit only. Due to technological issues, 14 extra home visits were provided to eLiFE participants, with two participants requiring more than one extra visit.

#### Activities Performed

At T2, participants reported whether and which of 25 possible activities they had performed (Supplementary Figure 2). On average, 9.1 (SD 5.1) activities were reported for eLiFE and 10.0 (SD 5.8) activities for aLiFE. eLiFE participants tended to select fewer activities but at more challenging levels, while aLiFE participants trained at all levels of difficulty. The “one-leg stand” was the most reported activity (*n* = 67) in both intervention groups, followed by “stair climbing” and “tandem walk” (both *n* = 58). The least reported activity was square jumping on one leg (*n* = 4).

#### Adherence

During the intervention period, 47 participants reported full adherence (16 eLiFE, 11 aLiFE, 20 controls), 88 reported partial adherence (28 eLiFE, 32 aLiFE, 28 controls), and 29 were non-adherent to their intervention (10 eLiFE, 12 aLiFE, 7 controls). During the unsupervised follow-up period, 34 participants reported full adherence (10 eLiFE, 7 aLiFE, 17 controls), 73 reported partial adherences (26 eLiFE, 25 aLiFE, 22 controls), and 40 were non-adherent to their intervention (10 eLiFE, 20 aLiFE, 10 controls). At T2 and T3, the participants' (*n* = 137) EARS scores were on average 12.20 (SD 2.29, range 6–18) for eLiFE; 11.85 (SD 2.44, range 6–18) for aLiFE; and 11.61 (SD 2.07, range 7–19) for controls. Although the adherence reporting was not set up as a motivating factor, participants reported it as motivating.

‘*more like a thing about “you're in a project”, and to note whether you've done anything or everything, so just a reminder from you'* (aLiFE participant, Trondheim)

#### Satisfaction

Participants' satisfaction with aLiFE and eLiFE is shown in Table 2. For habit formation (SRBAI), the scores at T2 and T3 were similar (4.57, SD 1.39, and 4.65, SD 1.38) indicating that habits formed during the first period (T1–T2) were sustained at follow-up (T3) (Supplementary Table 4). Some participants in the focus groups reported that many activities had become habitual, whereas some still required active thought and tangible reminders.

“*I don't even have to think of it. The moment I grab my toothbrush, I do one leg stance. That really became automatic.”* (eLiFE participant, Amsterdam)

“*I don't know, tandem walk through the hall and things like that, they would not fit into my daily routine, so I perform these exercises during my daily workout.”* (aLiFE participant, Stuttgart)

**Table 2:** Feasibility and acceptability of the technology delivered eLiFE and the traditional aLiFE version of the intervention.

**Elements of the intervention**	Range^*^	**6 months (T2)**		**12 months (T3)**	
		**aLiFE**	**eLiFE**	**aLiFE**	**eLiFE**
aLiFE Manual, mean (SD)	1–7	5.6 (1.1)	–	5.3 (1.2)	–
aLiFE planning and monitoring forms, mean (SD)	1–21	14.2 (13.7)	–	12.3 (4.2)	–
eLiFE virtual trainer, mean (SD)	1–7	–	5.1 (1.4)	–	5.2 (1.4)
eLiFE planning and messaging, mean (SD)	1–28	–	18.9 (4.3)	–	17.7 (14.4)
Perceived improvements in strength, balance and physical activity, mean (SD)	1–28	16.7 (2.2)	16.8 (2.2)	16.1 (2.7)	16.3 (2.4)
Helpfulness of the personal instructor, mean (SD)	1–7	6.5 (0.7)	6.2 (1.1)	6.4 (1.0)	5.95 (1.04)
Ease of performing activities, mean (SD)	1–7	4.6 (1.5)	5.0 (1.3)	4.7 (1.4)	5.2 (1.3)
Safety of activities, mean (SD)	1–7	5.8 (1.5)	6.1 (1.1)	5.8 (1.7)	5.9 (1.2)
Integration of activities into daily life, mean (SD)	1–7	4.7 (1.5)	4.9 (1.5)	4.6 (1.3)	5.1 (1.3)

* *With maximum score as the best score*.

The human factor, using instructors in both groups, were highly valued by the participants.

“*They* [instructors] *always showed it and demonstrated it and we always did it together…, so that was really ideal.”* (eLiFE participant, Stuttgart)

#### Adverse Events

During the 12-month trial period, twelve negative events were reported, with nine adverse reactions to the intervention and three falls during testing. The nine adverse reactions were classified as mild/moderate of which seven were pain/soreness, one a swollen knee, and one leg stiffness/tiredness. In addition, the subjects reported a further 19 serious adverse events as well as 28 other events, all of which were unrelated to the intervention.

#### eLiFE Usage

**Figure 2:**
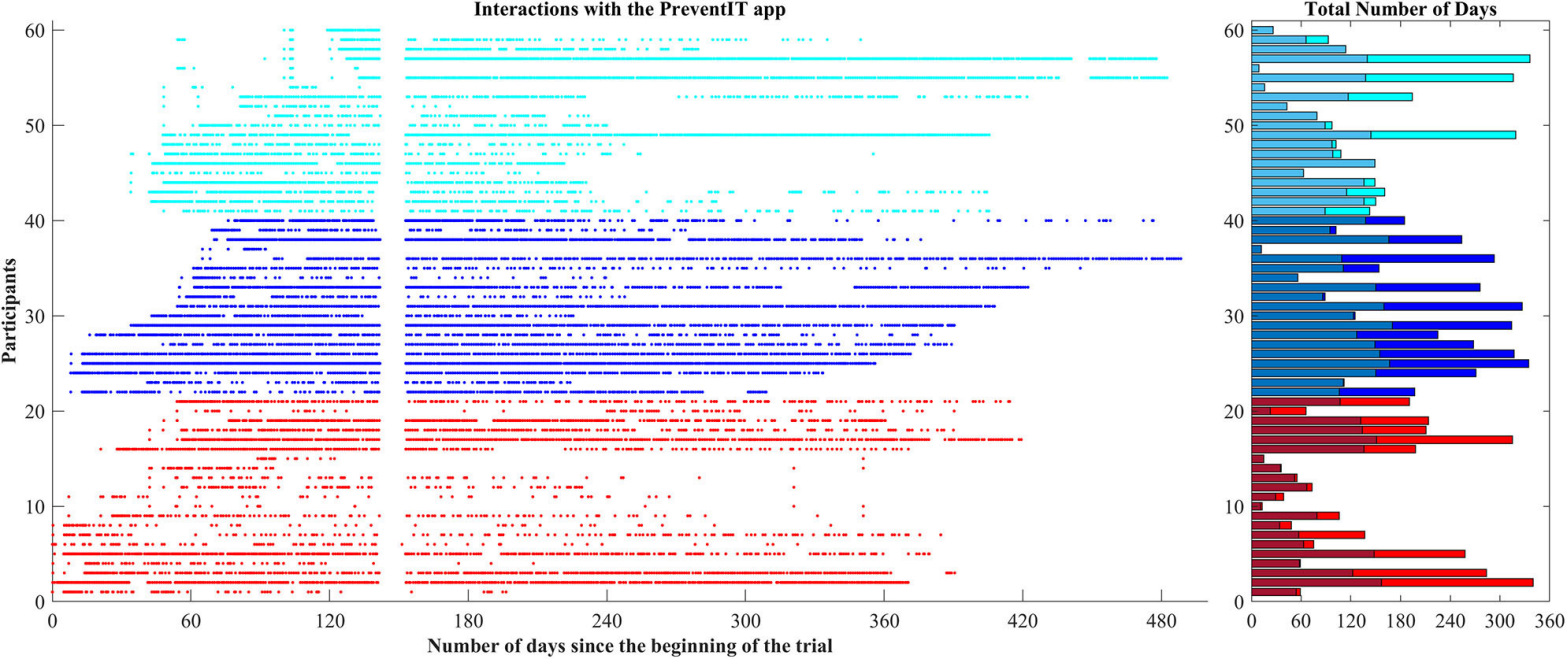
eLiFE participants' interactions with the app.

During the 12-month trial period, 58 out of 61 eLiFE participants used the application for on average 179.6 days (range 1–362 days), with 129.8 usage days until T2, and 49.7 usage days between T2 and T3 (Figure 2). Nineteen participants stopped using the application after T2. Over the 12 month period eLiFE participants selected on average 10.7 activities (range 1–25), and made on average 2.4 upgrades to more challenging activities (range 0–13).

#### eLiFE Acceptability

The mean SUS score for eLiFE participants was 62.04 ± 15.75, range 25–100 at T2, and 60.54 ± 15.11 range 27.5–90, at T3. The mean value of the TSQ-WT was 73.4 out of 120 (±15.42, range 36–100) at T2 and 71.05/120 (±16.20, range 40–104) at T3. The PreventIT system was, based on the scores of these two tests, regarded around average in terms of usability, which was also reflected in the focus group data, with some frustrations with the technology, but generally happiness with the overall concept:

“*I think that overall the technology aspect was not mature enough.”* (eLiFE participant, Stuttgart)

“*The idea is very good of course.”* (eLiFE participant, Amsterdam)

### Estimates of Change

No significant differences in change between the three groups were found for the primary outcome measures, LLFDI consisting of six variables and the physical behavior complexity metric (*p* > 0.01), or secondary outcomes (*p* > 0.01) (Supplementary Table 1). For several of the outcomes, all groups showed improvements over time (Supplementary Tables 2, 3, and Supplementary Figure 3).

### Health Economic Evaluation

Although there was no significant difference in mean 12-month costs per participant, the intervention groups showed lower overall costs (aLiFE vs. control: €-116, 95% CI: −1,340 to 1,108, eLiFE vs. control: €-25, 95% CI: −1,173 to 1,123, see Table 3). Regarding the QALYs, aLiFE was associated with more QALYs (0.0060, 95% CI: −0.0119 to 0.0239) while eLiFE was associated with lower (−0.0063, 95% CI: −0.0254 to 0.0127), but both differences were not statistically significant. Therefore, compared to control, aLiFE was associated with lower costs and more QALYs and at the thresholds considered, the NHBs were positive (0.0099 and 0.0089) and the probability of being cost-effective was 66.3 and 68.2% (Supplementary Figure 1), whereas for eLiFE, the incremental NHBs were both negative.

**Table 3:** Costs, EQ-5D scores, QALYs, and cost-effectiveness results.

	**aLiFE (*****n*** **=** **59)**		**eLiFE (*****n*** **=** **61)**		**Control (*****n*** **=** **60)**	
	**Mean (SE)**	**95% CI**	**Mean (SE)**	**95% CI**	**Mean (SE)**	**95% CI**
Total costs	1,616 (330)	951 to 2,281	1,764 (269)	1,223–2,306	1,729 (540)	643–2,816
Incremental costs^a^	−116	−1,340 to 1,108	−25	−1,173 to 1,123	–	
Baseline EQ-5D	0.8717 (0.0189)	0.8338 to 0.9096	0.8850 (0.0123)	0.8604 to 0.9097	0.8973 (0.0192)	0.8589 to 0.9357
6-month EQ-5D	0.8949 (0.0159)	0.8630 to 0.9268	0.8849 (0.0185)	0.8479 to 0.9219	0.9068 (0.0203)	0.8662 to 0.9474
12-month EQ-5D	0.9090 (0.0125)	0.8839 to 0.9341	0.9004 (0.0169)	0.8665 to 0.9344	0.9156 (0.0161)	0.8834 to 0.9479
Total QALYs	0.8926 (0.0142)	0.8641 to 0.9211	0.8888 (0.0149)	0.8589 to 0.9187	0.9066 (0.0181)	0.8703 to 0.9429
Incremental QALYs^b^	0.0060	−0.0119 to 0.0239	−0.0063	−0.0254 to 0.0127	–	
ICER (€/QALY) (intervention vs. control)	Index	–	Comparator			
**Incremental NHB at**						
30,000 €/QALY	0.0099		−0.0071		–	
40,000 €/QALY	0.0089		−0.0069		–	
**probability pf cost-effectiveness at**						
30,000 €/QALY	66.3%		–		–	
40,000 €/QALY	68.2%		–		–	

a *Costs adjusted for baseline resource use, age and sex, showing differences in costs between the intervention group and control.*

b *QALYs adjusted baseline HRQoL, age and sex, showing difference in QALYs between the intervention group and control*.

## Discussion

The PreventIT feasibility trial set out to evaluate aLiFE and eLiFE in young seniors, including participation and adherence, feasibility and usability, and acceptability of smartphone application-based intervention delivery. Findings aim to guide future definitive trials in this important target group for prevention of functional decline at later age. Recruitment of this target group and the interventions were feasible concerning intervention uptake and satisfaction. The behavior of participants changed during the 6 month lifestyle-integrated intervention programme, with more challenging everyday routines. All three groups improved in some of the clinical outcome measures over time, without significant group differences in primary or secondary outcomes. This may suggest that, improved function and behavioral change can be achieved in different ways. Furthermore, participants were satisfied with both the technology-based version and the traditional delivered paper-based version, but the “human element” of involving an instructor was valued as highly motivating. To increase satisfaction and uptake, future trials could explore further personalisation of the interventions.

An extensive protocol for screening and testing of participants was applied. The majority of participants completed all trial assessments, with 87% completing the 6-month post-test and 77% completing the 12-month follow-up assessment. In contrast, there was a higher number of withdrawals and dropouts during initial screening and testing. To avoid unnecessary burden on participants, assessments, and intervention methodologies should be kept as brief as possible.

In addition to the relatively high completion rate and low number of drop outs, the majority of participants answered positively concerning adherence, although many reported not doing as much activities as they had planned. Additional home visits were required due to technical issues with the technology system during the intervention period, which improved based on the participants' feedback. The short period available for developing and testing the technology in this project may explain several of the technical difficulties the participants experienced. Despite the immature technology, most participants liked the overall concept and continued using the system after the active intervention period. With further improvements, a system using smartwatches and smartphones to deliver an intervention to this target population is realistic (9). The higher system use during the initial active intervention period could indicate that participants need the system when learning the concept, or illustrate that interest in new applications is highest in the beginning^1^. Further development of the application should therefore include tailoring the intervention to different phases of use.

An important strength of the PreventIT project has been the development and testing of explicit links between behavioral change techniques and intervention components, which has been a major gap in previous studies. Furthermore, the systematic collection of adherence data, which is feasible when using smartphones as platform for delivering the intervention, is another strength. Finally, the use of lifestyle-integration of the intervention may have increased adherence to exercise over longer periods.

We included relatively healthy participants, with all groups showing improvements over time regardless of group allocation. For future improvements of the interventions, the dosage and intensity for a rather fit group should be considered, along with the possible benefit to the control group, becoming aware of their health situation because of extensive assessments, along with learning how to be more physically active. The monthly question regarding adherence levels may have served as a reminder to be active in all three groups, rather than being an observational outcome measure only. Progress on difficulty level was part of the interventions, but how and when to progress should be evaluated in future trials, as few participants progressed through all available difficulty levels of the activities. Furthermore, complexity in behavior as an experimental outcome measure was promising, and has the potential to detect changes even in high functioning individuals—research should explore this further in future trials.

For the purpose of estimating cost-effectiveness, a self-reported healthcare resource use questionnaire was employed. GP visits, inpatient days, and physiotherapy visits were effectively collected, demonstrating the feasibility. Only small differences in QALYs between intervention and control groups were observed, most likely due to high baseline EQ-5D-5L scores and thereby poor discriminative ability due to ceiling effects (37). Alternative tools should be considered for future trials if similar target groups are included.

### Limitations

An important study limitation is the inclusion of a relatively healthy group of young seniors, which might not represent the general population of that age group. Participants were not excluded based on their IT literacy, which might have influenced the results of the study. We did however provide participants without any smartphone experience one extra home visit, with information on how to use a smartphone prior to starting the home visits in week one. Further research is needed to involve participants at medium risk for long-term functional decline when delivering physical activity promotion interventions (17). For the majority of participants in our study, the exercise activities might not have been challenging enough and not practiced for long enough. Nevertheless, all groups showed improvements over time, suggesting that even in this target group, health benefits can be achieved. Most importantly, this feasibility trial reflects the challenge of reaching and engaging those people at risk, who would benefit most from the intervention.

## Conclusion

In conclusion, the PreventIT feasibility RCT has shown that it is possible to deliver a 6 month lifestyle-integrated intervention programme to young seniors. Feasibility, usability and acceptability of eLiFE using an ICT platform, combining technology with behavior change techniques, was as successful as delivering the intervention using traditional paper-based manuals. This intervention was shown to have the potential to change participants' physical activity behavior and make daily routines more challenging. Participants liked the concept and many of them were still using either the application or the manual during the unsupervised follow-up period. Possibilities for further development of interventions targeting young seniors include longer follow up periods, specific recruitment strategies for those at higher risk for future functional decline and further adaptations of the content of the intervention delivered.

## Data Availability Statement

The datasets presented in this article are not readily available because the datasets for this study are not open accessible to all, but only shared between project partners due to ethical approval guidelines in the three clinical sites conducting the multicenter trial, and cannot be shared until 10 years after the end of the project. Requests to access the datasets should be directed to kristin.taraldsen@ntnu.no.

## Ethics Statement

The studies involving human participants were reviewed and approved by the ethical committees in Norway (REK midt, 2016/1891), Stuttgart (registration number 770/2016BO1), and Amsterdam [METc VUmc registration number 2016.539 (NL59977.029.16)]. The patients/participants provided their written informed consent to participate in this study.

## Author Contributions

All authors contributed to the planning, conduct, and reporting of the work described in the article. JH was responsible for the overall content as guarantor. The corresponding author attest that all listed authors meet authorship criteria and that no others meeting the criteria have been omitted.

## Conflict of Interest

SM and LC own a share in the spin-off company of the University of Bologna, mHealth Technologies srl. AZ was funded by the Doxee s.p.a. during the conduction of the trial, and by CINECA after the end of the project. The remaining authors declare that the research was conducted in the absence of any commercial or financial relationships that could be construed as a potential conflict of interest.

## Footnotes

^1^Available online at: https://www.gartner.com/en/research/methodologies/gartner-hype-cycle.
