# Supplementary figures and images for: Digital Technology to Deliver a Lifestyle-Integrated Exercise Intervention in Young Seniors—The PreventIT Feasibility Randomized Controlled Trial
Source: Front Digit Health. 2020 Jul 31;2:10. doi: 10.3389/fdgth.2020.00010 (PMC8521904; doi:10.3389/fdgth.2020.00010)

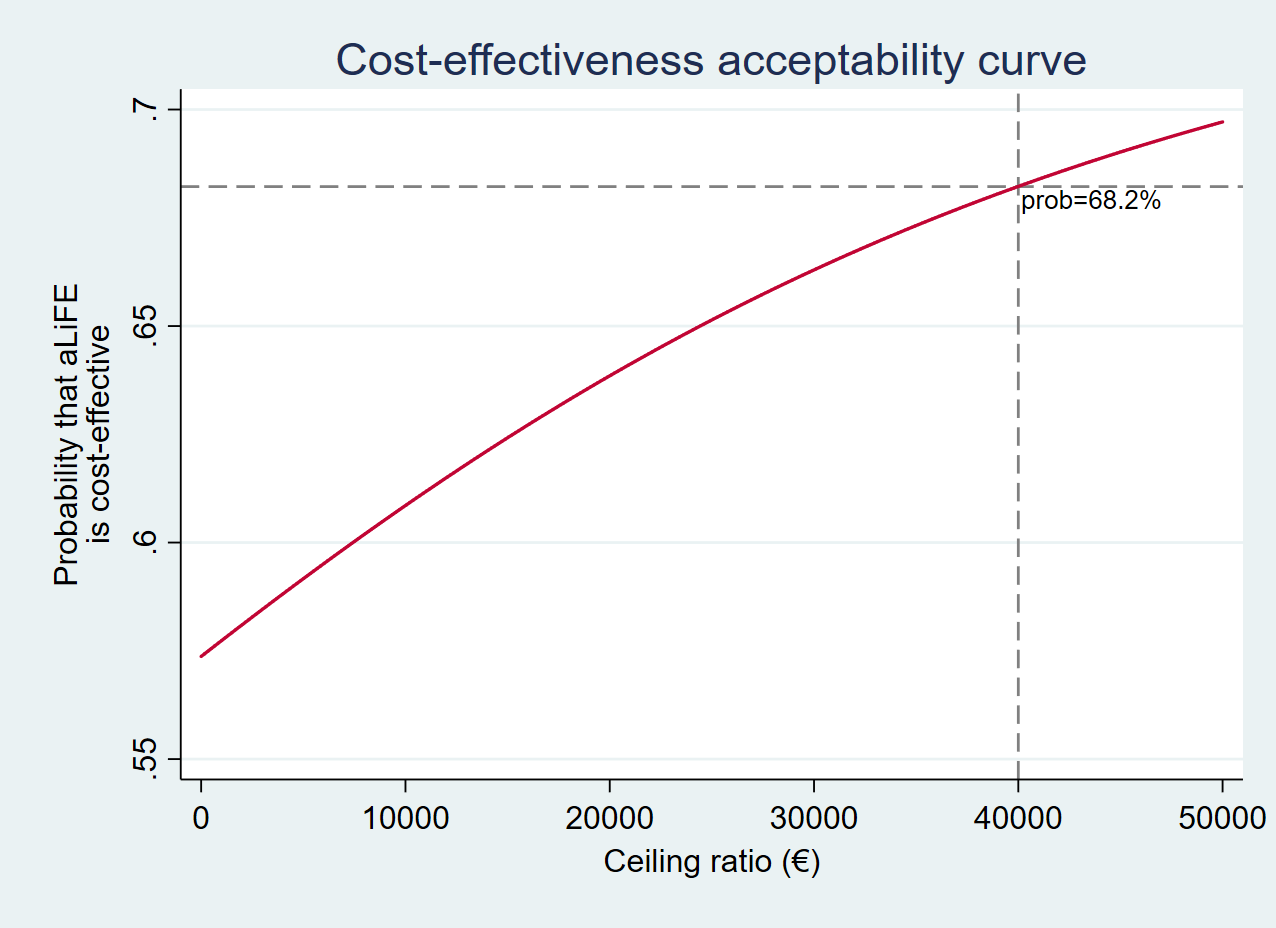

Supplement: Supplementary file 1 [file Image_1.tif]

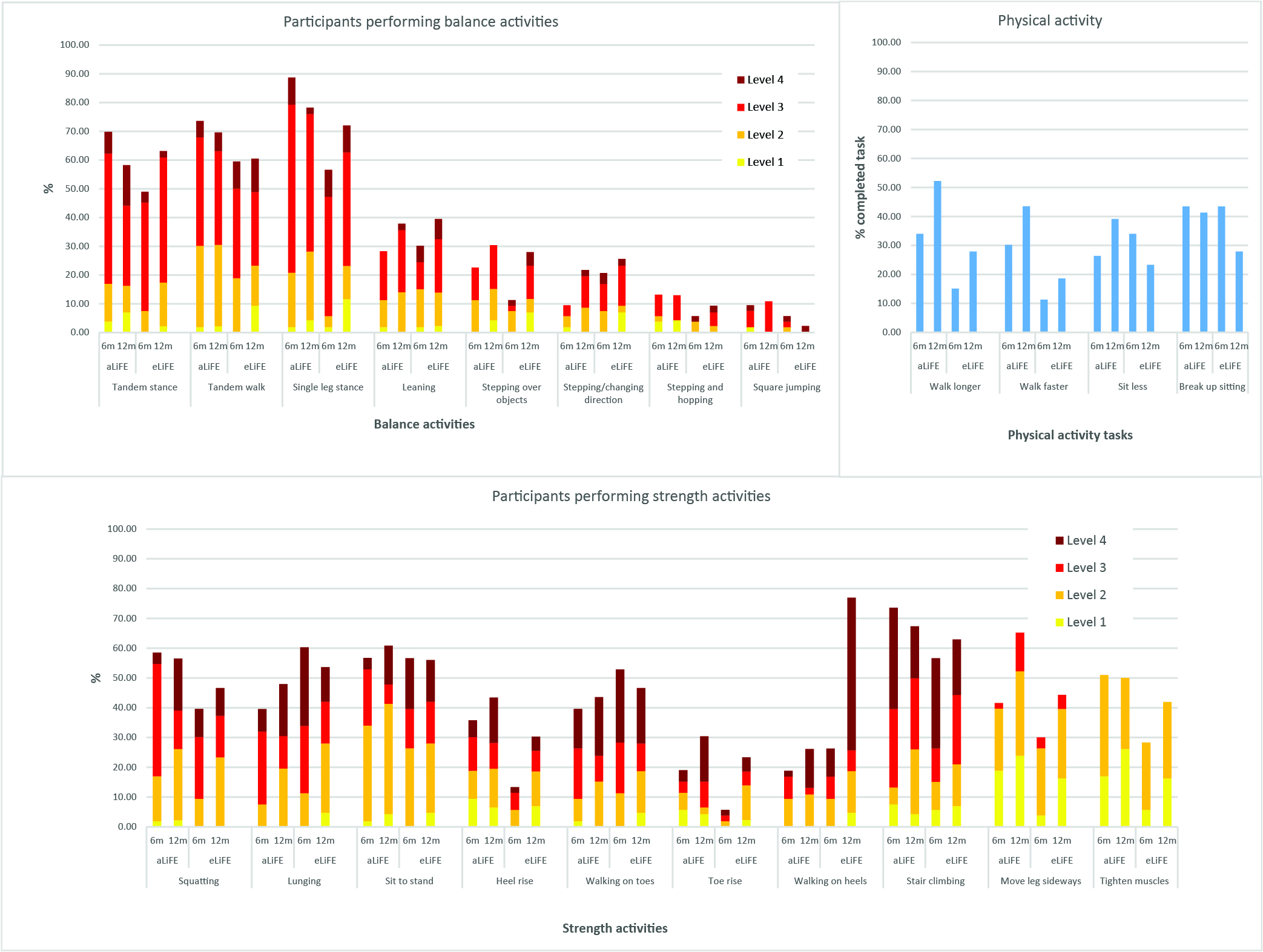

Supplement: Supplementary file 2 [file Image_2.jpeg]

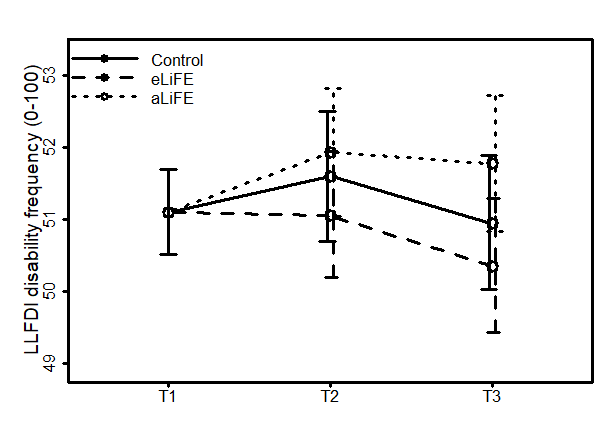

Supplement: Supplementary file 3 [file Image_3.tiff]

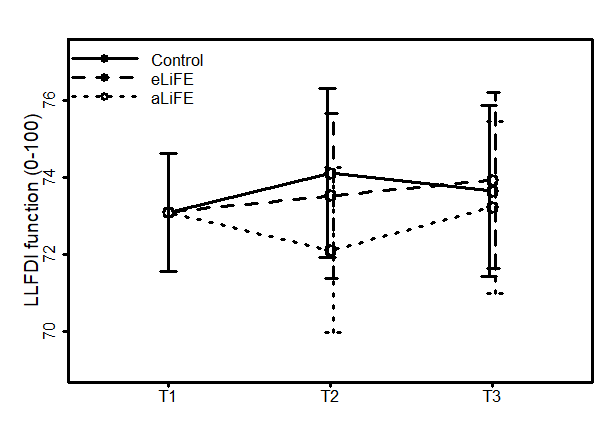

Supplement: Supplementary file 4 [file Image_4.tiff]

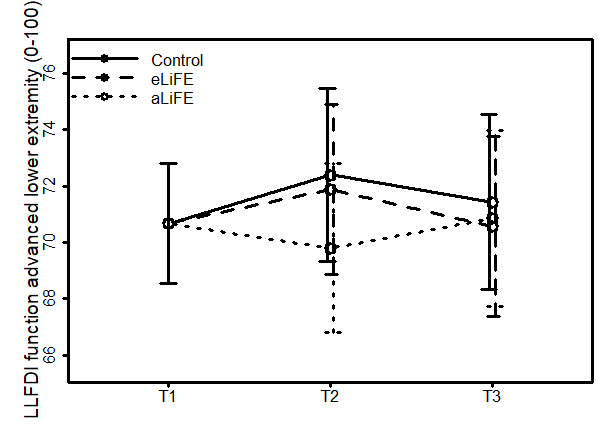

Supplement: Supplementary file 5 [file Image_5.tiff]

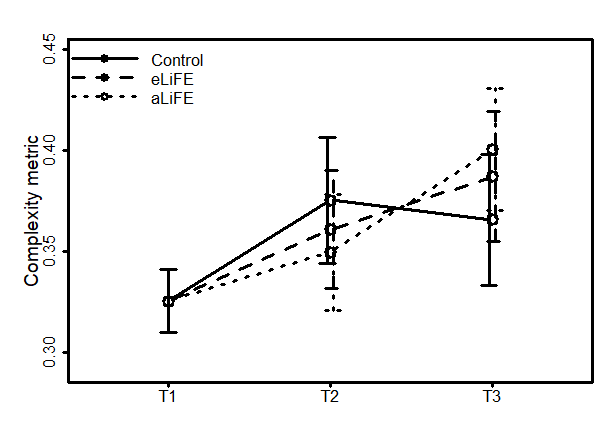

Supplement: Supplementary file 6 [file Image_6.tiff]
