# Supplementary material for: Digital Technology to Deliver a Lifestyle-Integrated Exercise Intervention in Young Seniors—The PreventIT Feasibility Randomized Controlled Trial
Source: Front Digit Health. 2020 Jul 31;2:10. doi: 10.3389/fdgth.2020.00010 (PMC8521904; doi:10.3389/fdgth.2020.00010)
Supplement: Supplementary file 7 [file Data_Sheet_1.docx]

Supplementary Material

# Supplementary Tables

**eTable 1.** Estimated between group differences in primary and secondary outcomes. *) Non-parametric tests, with the value of the test statistic given in the “Mean” column, otherwise results from linear mixed models with means and 95% CIs back-transformed to original scale in the case of log-transformed data (LLFDI disability frequency).

|  | eLiFE vs controls | | | | | | aLiFE vs controls | | | | | |
| --- | --- | --- | --- | --- | --- | --- | --- | --- | --- | --- | --- | --- |
|  | T2-T1 | | | T3-T1 | | | T2-T1 | | | T3-T1 | | |
|  | Mean | 95% CI | p-value | Mean | 95% CI | p-value | Mean | 95% CI | p-value | Mean | 95% CI | p-value |
| Primary outcomes: | | | | | | | | | | | | |
| LLFDI dis.freq. | -0.55 | (-1.68,0.59) | 0.347 | -0.60 | (-1.80,0.59) | 0.324 | 0.33 | (-0.78,1.50) | 0.569 | 0.83 | (-0.32,2.03) | 0.171 |
| LLFDI dis.lim. *) | 0.49 |  | 0.871 | 0.45 |  | 0.431 | 0.44 |  | 0.300 | 0.44 |  | 0.311 |
| LLFDI Function | -0.59 | (-3.16,1.98) | 0.652 | 0.28 | (-2.42,2.98) | 0.839 | -2.01 | (-4.57,0.56) | 0.126 | -0.43 | (-3.09,2.24) | 0.754 |
| LLFDI func.UE *) | 0.47 |  | 0.627 | 0.47 |  | 0.639 | 0.47 |  | 0.562 | 0.47 |  | 0.634 |
| LLFDI func.BLE *) | 0.46 |  | 0.507 | 0.55 |  | 0.394 | 0.41 |  | 0.107 | 0.48 |  | 0.701 |
| LLFDI func.ALE | -0.51 | (-4.14,3.12) | 0.783 | -0.86 | (-4.68,2.96) | 0.660 | -2.60 | (-6.22,1.03) | 0.160 | -0.58 | (-4.34,3.19) | 0.763 |
| Complexity (unitless) | -0.01 | (-0.06,0.03) | 0.501 | 0.02 | (-0.02,0.07) | 0.354 | -0.03 | (-0.07,0.02) | 0.228 | 0.03 | (-0.01,0.08) | 0.123 |
| Secondary outcomes: | | | | | | | | | | | | |
| Percentage Walking | -1.0 | (-2.7,0.6) | 0.244 | -1.3 | (-3.2,0.6) | 0.180 | -0.4 | (-2.1,1.2) | 0.607 | -0.4 | (-2.4,1.4) | 0.643 |
| Maximum Walking bout length (minutes) | -190 | (-414.6,12.8) | 0.074 | -115.7 | (-336.0,93.1) | 0.279 | -106.8 | (-335.6,119.4) | 0.342 | -44.8 | (-265.4,164.4) | 0.682 |
| Cadence Maximum Time Walking bout (steps/min) *) | 0.5 |  | 0.977 | 0.59 |  | 0.197 | 0.53 |  | 0.586 | 0.56 |  | 0.323 |
| CBMS sum score, 0-66 *) | 0.53 |  | 0.653 | 0.58 |  | 0.204 | 0.54 |  | 0.521 | 0.58 |  | 0.170 |
| Completed 8-level balance scale, 0-8 *) | 0.55 |  | 0.340 | 0.58 |  | 0.148 | 0.62 |  | 0.019 | 0.59 |  | 0.097 |
| Gait speed, 4m preferred, m/sec | -0.03 | (-0.08,0.03) | 0.378 | -0.05 | (-0.11,0.01) | 0.098 | -0.02 | (-0.08,0.03) | 0.402 | -0.03 | (-0.09,0.03) | 0.328 |
| Gait speed, 7m preferred, m/sec | 0.00 | (-0.06,0.05) | 0.951 | -0.05 | (-0.11,0.01) | 0.114 | 0.02 | (-0.04,0.08) | 0.460 | 0.01 | (-0.05,0.07) | 0.760 |
| Gait speed, 7m fast, m/sec | -0.01 | (-0.11,0.09) | 0.796 | -0.03 | (-0.13,0.08) | 0.635 | 0.03 | (-0.07,0.13) | 0.510 | 0.02 | (-0.09,0.12) | 0.758 |
| Grip strength, kg | -0.5 | (-2.3,1.2) | 0.553 | 0.3 | (-1.5,2.2) | 0.732 | -0.9 | (-2.6,0.9) | 0.320 | 0.3 | (-1.5,2.1) | 0.765 |
| STS sec *) | 0.5 |  | 0.970 | 0.42 |  | 0.218 | 0.50 |  | 0.984 | 0.45 |  | 0.409 |
| MOCA sum score, 0-30 *) | 0.54 |  | 0.486 | 0.57 |  | 0.219 | 0.58 |  | 0.161 | 0.59 |  | 0.134 |
| FESI sum, 7-28 *) | 0.50 |  | 0.961 | 0.40 |  | 0.106 | 0.43 |  | 0.219 | 0.42 |  | 0.146 |
| CESD total score, 0-60 *) | 0.53 |  | 0.578 | 0.47 |  | 0.604 | 0.46 |  | 0.431 | 0.41 |  | 0.144 |
| SF12 total Sum *) | 0.64 |  | 0.013 | 0.53 |  | 0.602 | 0.54 |  | 0.459 | 0.55 |  | 0.364 |
| PCS *) | 0.51 |  | 0.919 | 0.50 |  | 0.968 | 0.59 |  | 0.113 | 0.50 |  | 0.939 |
| MCS *) | 0.46 |  | 0.501 | 0.44 |  | 0.348 | 0.46 |  | 0.480 | 0.47 |  | 0.606 |
| EQ5D *) | 0.50 |  | 0.947 | 0.51 |  | 0.818 | 0.52 |  | 0.721 | 0.57 |  | 0.209 |

**Table e1 continues…**

|  | aLiFE vs eLiFE | | | | | |
| --- | --- | --- | --- | --- | --- | --- |
|  | T2-T1 | | | T3-T1 | | |
|  | Mean | 95% CI | p-value | Mean | 95% CI | p-value |
| Primary outcomes: | | | | | | |
| LLFDI dis.freq. | 0.88 | (-0.24 to 2.01) | 0.125 | 1.42 | (0.23 to 2.63) | 0.020 |
| LLFDI dis.lim. *) | 0.44 |  | 0.322 | 0.50 |  | 0.993 |
| LLFDI Function | -1.42 | (-3.95 to 1.11) | 0.273 | -0.71 | (-3.43 to 2.01) | 0.610 |
| LLFDI func.UE *) | 0.50 |  | 0.958 | 0.50 |  | 0.983 |
| LLFDI func.BLE *) | 0.44 |  | 0.278 | 0.42 |  | 0.186 |
| LLFDI func.ALE | -2.09 | (-5.66 to 1.49) | 0.252 | 0.28 | (-3.56 to 4.12) | 0.887 |
| Complexity | -0.01 | (-0.05 to 0.03) | 0.582 | 0.01 | (-0.03 to 0.06) | 0.551 |
| Secondary outcomes: | | | | | | |
| Percentage Walking | 0.6 | (-1.1 to 2.1) | 0.497 | 0.8 | (-1 to 2.6) | 0.359 |
| Maximum Walking bout length (minutes) | 83.2 | (-103.0 to 275.9) | 0.381 | 70.9 | (-129.7 to 268.5) | 0.479 |
| Cadence Maximum Time Walking bout *) | 0.52 |  | 0.766 | 0.47 |  | 0.698 |
| CBMS sum score, 0-66 *) | 0.52 |  | 0.682 | 0.52 |  | 0.766 |
| Completed 8-level balance scale, 0-8 *) | 0.58 |  | 0.145 | 0.51 |  | 0.897 |
| Gait speed, 4m preferred, m/sec | 0.00 | (-0.06 to 0.06) | 0.965 | 0.02 | (-0.04 to 0.08) | 0.492 |
| Gait speed, 7m preferred, m/sec | 0.02 | (-0.03 to 0.08) | 0.417 | 0.06 | (0.00 to 0.12) | 0.061 |
| Gait speed, 7m fast, m/sec | 0.05 | (-0.05 to 0.14) | 0.352 | 0.04 | (-0.06 to 0.15) | 0.438 |
| Grip strength, kg | -0.4 | (-2.1 to 1.4) | 0.684 | 0.0 | (-1.9 to 1.8) | 0.962 |
| STS sec *) | 0.50 |  | 0.997 | 0.53 |  | 0.653 |
| MOCA sum score, 0-30 *) | 0.54 |  | 0.456 | 0.52 |  | 0.790 |
| FESI sum, 7-28 *) | 0.44 |  | 0.233 | 0.51 |  | 0.835 |
| CESD total score, 0-60 *) | 0.43 |  | 0.199 | 0.44 |  | 0.355 |
| SF12 total Sum *) | 0.40 |  | 0.079 | 0.53 |  | 0.676 |
| PCS *) | 0.57 |  | 0.202 | 0.51 |  | 0.907 |
| MCS *) | 0.50 |  | 0.985 | 0.52 |  | 0.716 |
| EQ5D *) | 0.52 |  | 0.669 | 0.56 |  | 0.354 |

Note: Subgroup analysis dependent on risk of functional decline or reported adherence did not reveal any significant group differences and are not reported.

LLFDI=Late Life Function and Disability Index; The LLFDI variables included: dis.freq=Disability frequency score; dis.lim=Disability limitation score; Function=total score function; func.UE=Function in Upper Extremity score; func.BLE=Function in Basic Lower Extremity score; func.ALE=Function in Advanced Lower Extremity score. Complexity=Wplzc MEAN as a measure representing behavioural complexity; Percentage Walking=Percentage of walking per day (minutes as unit for walking and percentage averaged over the collected days); Maximum Walking bout length=the maximum walking bout length registered over the collected period; Cadence Maximum Time Walking bout=cadence during the maximal continuous walking episode recorded; CBMS=Community Balance and Mobility Scale; STS=5 times sit-to-stand test; MOCA=Montreal Cognitive Assessment; FESI=Fear of falling Scale International; CESD=the center for epidemiologic studies depression scale ; SF-12=short form 12 health survey; PCS=Physical Component Summary; MCS=Mental Component Summary; EQ5D=5-level EuroQol 5-dimension instrument

**eTable 2.** Primary and secondary outcome measures for all three groups (eLiFE, aLiFE, and controls) at T1, T2, and T3

|  | T1 | | | T2 | | | T3 | | | | |
| --- | --- | --- | --- | --- | --- | --- | --- | --- | --- | --- | --- |
|  | eLiFE | aLiFE | controls | eLiFE | aLiFE | controls | eLiFE | | aLiFE | controls | |
| Primary outcomes: | | | | | | | |  |  |  |  |
| LLFDI dis.freq. | 49.48 (47.56 - 52.96) | 52.23 (48.83 - 54.49) | 51.17 (48.19 - 53.91) | 50.15 (48.19 - 52.96) | 51.52 (50.15 - 55.3) | 51.52 (48.35 - 54.49) | 49.48 (46.93 - 51.88) | | 51.17 (48.83 - 54.3) | 51.52 (48.67 - 53.71) |  |
| LLFDI dis.lim. *) | 80.03 (72.59 - 100) | 83.44 (72.59 - 100) | 80.03 (71.33 - 89.31) | 80.03 (74 - 100) | 83.44 (72.59 - 100) | 86.38 (74 - 100) | 83.44 (72.59 - 100) | | 83.44 (75.63 - 100) | 89.31 (75.63 - 100) |  |
| LLFDI Function | 73.08 (10.58) | 73.70 (12.88) | 73.30 (13.03) | 73.19 (11.29) | 72.19 (12.77) | 74.15 (13.62) | 73.83 (11.87) | | 72.90 (13.44) | 73.59 (13.31) |  |
| LLFDI func.UE *) | 88 (82 - 100) | 88 (82 - 100) | 82 (73.94 - 100) | 88 (82 - 100) | 88 (77.5 - 100) | 88 (74.83 - 97) | 88 (82 - 100) | | 88 (82 - 100) | 88 (80.88 - 91) |  |
| LLFDI func.BLE *) | 81.17 (77.17 - 88.02) | 88.02 (72.08 - 100) | 81.17 (69.83 - 100) | 81.17 (74.31 - 100) | 81.17 (74.31 - 100) | 81.17 (74.31 - 100) | 88.02 (79.17 - 100) | | 81.17 (77.17 - 100) | 88.02 (71.62 - 100) |  |
| LLFDI func.ALE | 70.6 (14.96) | 70.39 (15.84) | 72.04 (16.59) | 71.24 (17.50) | 68.96 (16.70) | 73.13 (18.21) | 70.49 (16.63) | | 69.38 (17.22) | 72.02 (18.82) |  |
| Complexity | 0.33 (0.13) | 0.33 (0.12) | 0.32 (0.13) | 0.36 (0.10) | 0.35 (0.09) | 0.38 (0.09) | 0.39 (0.11) | | 0.39 (0.11) | 0.37 (0.08) |  |
| Secondary outcomes: | | | | | | | |  |  |  |  |
| Percentage Walking | 14.8 (11.7 - 20.1) | 13.8 (11.0 - 18.6) | 15.9 (12.3 - 19.4) | 13.5 (10.6 - 16.4) | 13.2 (10.7 - 16.3) | 15.3 (11.6 - 18.1) | 14.4 (12.0 - 18.6) | | 14.5 (11.3 - 17.5) | 15.9 (12.5 - 18.3) |  |
| Maximum Walking bout length, min | 595 (340 - 996) | 584 (317 - 790) | 659 (369.5 – 859) | 517 (327 - 789.8) | 547 (376 - 1025) | 739 (423 - 1231) | 585 (336 - 776) | | 464 (324.2 - 783.2) | 592.5 (348.5 - 894.5) |  |
| Cadence Maximum Time Walking bout | 108 (100 - 117) | 111 (104 - 117) | 111 (107 - 117.5) | 111 (100.8 - 118) | 111 (106 - 122) | 113 (110 - 122) | 110 (99 - 115.5) | | 108.5 (101.2 - 116.2) | 108 (103 - 117) |  |
| CBMS sum score, 0-66 | 66.5 (60.8 - 74.2) | 67 (58 - 73.5) | 65.5 (59 - 72.2) | 74 (65 - 79) | 72 (62.5 - 79.8) | 73.5 (66 - 78) | 77 (67.5 - 82) | | 73 (62.8 - 82.2) | 73 (66 - 80) |  |
| Completed 8-level balance scale, 0-8 | 4 (4 - 4) | 4 (3 - 4) | 4 (4 - 4) | 4 (4 - 4) | 4 (4 - 5) | 4 (4 - 4) | 4 (4 - 4) | | 4 (4 - 4) | 4 (4 - 4.5) |  |
| Gait speed, 4m preferred, m/sec | 1.39 (0.21) | 1.40 (0.23) | 1.38 (0.19) | 1.37 (0.20) | 1.40 (0.24) | 1.42 (0.21) | 1.38 (0.18) | | 1.41 (0.21) | 1.43 (0.17) |  |
| Gait speed, 7m preferred, m/sec | 1.54 (0.25) | 1.51 (0.25) | 1.53 (0.23) | 1.49 (0.18) | 1.51 (0.24) | 1.51 (0.19) | 1.46 (0.17) | | 1.51 (0.23) | 1.52 (0.20) |  |
| Gait speed, 7m fast, m/sec | 2.04 (0.51) | 2.04 (0.48) | 2.10 (0.39) | 2.02 (0.37) | 2.07 (0.43) | 2.08 (0.30) | 2.01 (0.39) | | 2.07 (0.39) | 2.10 (0.32) |  |
| Grip strength, kg | 34.3 (11.6) | 35.5 (10.5) | 34.7 (12.2) | 33.4 (10.6) | 34.0 (11.6) | 33.4 (12.5) | 32.2 (11.2) | | 34.6 (11.9) | 31.5 (11.7) |  |
| STS sec | 10.52 (9.33 - 11.9) | 10.25 (8.88 - 11.78) | 9.85 (8.17 - 11.52) | 10.36 (8.75 - 12.08) | 9.76 (8.5 - 12) | 9.91 (8.12 - 11.85) | 9.64 (8.03 - 11.12) | | 9.68 (7.71 - 11.93) | 9.58 (7.24 - 12.34) |  |
| MOCA sum score, 0-30 | 27 (26 - 29) | 27 (26 - 29) | 27 (25 - 29) | 27 (26 - 29) | 28 (26.5 - 29) | 27 (26 - 29) | 28 (28 - 29) | | 29 (27 - 30) | 28 (26 - 29) |  |
| FESI sum, 7-28 | 8 (7 - 9) | 8 (7 - 9) | 8 (7 - 9) | 7 (7 - 9) | 7 (7 - 8) | 7 (7 - 9) | 7 (7 - 8.5) | | 7 (7 - 8) | 8 (7 - 9) |  |
| CESD total score, 0-60 | 7 (4 - 11) | 8 (3.5 - 13.5) | 6.5 (4 - 11) | 6 (3 - 11) | 6 (2 - 11) | 4 (2 - 8) | 5 (2 - 12) | | 4 (1.2 - 8) | 5 (2.8 - 8.2) |  |
| SF12 total Sum | 39 (38 - 42) | 40 (38 - 42) | 40 (38 - 43) | 42 (40 - 43) | 41 (40 - 42) | 41 (39 - 42) | 41 (39.5 - 42) | | 41.5 (39 - 43) | 41 (39 - 42) |  |
| PCS | 50.4 (43.3 - 56) | 50.7 (42.8 - 55) | 51.3 (45.4 - 54.8) | 51 (43.2 - 56.1) | 52.4 (47.3 - 54.8) | 51.6 (43.4 - 54.8) | 51.3 (46.8 - 56.4) | | 49.7 (43.5 - 55.1) | 53.5 (42.3 - 56.1) |  |
| MCS | 53.7 (47 - 58.7) | 53.9 (48.6 - 58.8) | 56.5 (50 - 58.4) | 56.9 (49.1 - 59) | 57.1 (50.9 - 60.1) | 57.3 (52.4 - 60.1) | 55.5 (51.2 - 58.5) | | 57.8 (52.1 - 59.2) | 57.3 (52.5 - 60) |  |
| EQ5D | 0.89 (0.82 - 0.95) | 0.92 (0.83 - 0.95) | 0.94 (0.89 - 0.95) | 0.92 (0.85 - 0.95) | 0.94 (0.85 - 1) | 0.94 (0.89 - 1) | 0.94 (0.86 - 0.99) | | 0.94 (0.85 - 0.99) | 0.94 (0.89 - 1) |  |

Note: mean and SD values provided for data on orginal scale, median and 25^th^ and 75^th^ percentile for transformed or non-parametric data

LLFDI=Late Life Function and Disability Index; The LLFDI variables included: dis.freq=Disability frequency score; dis.lim=Disability limitation score; Function=total score function; func.UE=Function in Upper Extremity score; func.BLE=Function in Basic Lower Extremity score; func.ALE=Function in Advanced Lower Extremity score. Complexity= Wplzc MEAN as a measure representing behavioural complexity; Complexity=Wplzc MEAN as a measure representing behavioural complexity; Percentage Walking=Percentage of walking per day (minutes as unit for walking and percentage averaged over the collected days); Maximum Walking bout length=the maximum walking bout length registered over the collected period; Cadence Maximum Time Walking bout=cadence during the maximal continuous walking episode recorded; CBMS=Community Balance and Mobility Scale; STS=5 times sit-to-stand test; MOCA=Montreal Cognitive Assessment; FESI=Fear of falling Scale International; CESD=the center for epidemiologic studies depression scale ; SF-12=short form 12 health survey; PCS=Physical Component Summary; MCS=Mental Component Summary; EQ5D=5-level EuroQol 5-dimension instrument

**eTable 3.** Estimated change in time for the primary outcomes. *) Non-parametric tests, with the value of the test statistic given in the “Mean” column, otherwise results from linear mixed models with means and 95% Cis back-transformed to original scale in the case of log-transformed data (LLFDI_dis_fre).

|  | T2 vs T1 | | | T3 vs T1 | | |
| --- | --- | --- | --- | --- | --- | --- |
|  | Mean | 95% CI | p-value | Mean | 95% CI | p-value |
| LLFDI dis.freq. | 0.42 | (-0.07,0.92) | 0.096 | -0.07 | (-0.59,0.45) | 0.784 |
| LLFDI dis.lim. *) | 0.45 |  | 0.003 | 0.45 |  | 0.010 |
| LLFDI Function | 0.14 | (-0.96,1.24) | 0.804 | 0.48 | (-0.67,1.64) | 0.409 |
| LLFDI func.UE *) | 0.26 |  | 0.181 | 0.29 |  | 0.682 |
| LLFDI func.BLE *) | 0.33 |  | 0.313 | 0.42 |  | 0.005 |
| LLFDI func.ALE | 0.67 | (-0.88,2.22) | 0.397 | 0.29 | (-1.33,1.92) | 0.724 |
| Complexity | 0.04 | (0.02,0.06) | 0.001 | 0.06 | (0.04,0.08) | <0.001 |

The LLFDI variables included: dis.freq=Disability frequency score; dis.lim=Disability limitation score; Function=total score function; func.UE=Function in Upper Extremity score; func.BLE=Function in Basic Lower Extremity score; func.ALE=Function in Advanced Lower Extremity score. Complexity= Wplzc MEAN as a measure representing behavioural complexity.

**eTable 4. SRBAI (Habit formation)**

|  | SRBAI T2 | | | | | SRBAI T3 | | | | |
| --- | --- | --- | --- | --- | --- | --- | --- | --- | --- | --- |
|  | n | Mean | SD | Min | max | n | Mean | SD | Min | max |
| Controls | 49 | 4.74 | 1.43 | 1.75 | 7 | 48 | 4.89 | 1.23 | 1.75 | 7 |
| eLiFE | 51 | 4.63 | 1.46 | 1.75 | 7 | 40 | 4.63 | 1.46 | 1.00 | 7 |
| aLiFE | 50 | 4.46 | 1.50 | 1.25 | 7 | 45 | 4.42 | 1.45 | 1.00 | 7 |
| **Total** | **150** | **4.57** | **1.39** | **1.25** | **7** | **133** | **4.65** | **1.38** | **1.00** | **7** |

# Supplementary Figures

The supplementary figures are uploaded separately. The figure captions are as follows:

**Supplementary Figure 1.** Cost-effectiveness acceptability curve for aLiFE

**Supplementary Figure 2.** Performance of activities

**Supplementary Figure 3.** Estimates of change for primary outcomes in the three groups

**Note** that the Supplementary Figure 3 is uploaded as four separate files, named as follows:

Supplementary Figure 3a

Supplementary Figure 3b

Supplementary Figure 3c

Supplementary Figure 3d
